# Deep learning prediction of non-perfused volume without contrast agents during prostate ablation therapy

**DOI:** 10.1007/s13534-022-00250-y

**Published:** 2022-11-08

**Authors:** Cameron Wright, Pietari Mäkelä, Alexandre Bigot, Mikael Anttinen, Peter J. Boström, Roberto Blanco Sequeiros

**Affiliations:** 1 Department of Urology, University of Turku and Turku University Hospital, Turku, Finland; 2 Department of Diagnostic Radiology, University of Turku and Turku University Hospital, Turku, Finland; 3 Profound Medical Corp., Mississauga, Canada

**Keywords:** High intensity focused ultrasound, Deep learning, Contrast-enhanced MRI, UNet model, Clinical trials-thermal ablation, Control systems engineering, Treatment optimization

## Abstract

The non-perfused volume (NPV) is an important indicator of treatment success immediately after prostate ablation. However, visualization of the NPV first requires an injection of MRI contrast agents into the bloodstream, which has many downsides. Purpose of this study was to develop a deep learning model capable of predicting the NPV immediately after prostate ablation therapy without the need for MRI contrast agents. A modified 2D deep learning UNet model was developed to predict the post-treatment NPV. MRI imaging data from 95 patients who had previously undergone prostate ablation therapy for treatment of localized prostate cancer were used to train, validate, and test the model. Model inputs were T1/T2-weighted and thermometry MRI images, which were always acquired without any MRI contrast agents and prior to the final NPV image on treatment-day. Model output was the predicted NPV. Model accuracy was assessed using the Dice-Similarity Coefficient (DSC) by comparing the predicted to ground truth NPV. A radiologist also performed a qualitative assessment of NPV. Mean (std) DSC score for predicted NPV was 85% ± 8.1% compared to ground truth. Model performance was significantly better for slices with larger prostate radii (> 24 mm) and for whole-gland rather than partial ablation slices. The predicted NPV was indistinguishable from ground truth for 31% of images. Feasibility of predicting NPV using a UNet model without MRI contrast agents was clearly established. If developed further, this could improve patient treatment outcomes and could obviate the need for contrast agents altogether.

*Trial Registration Numbers* Three studies were used to populate the data: NCT02766543, NCT03814252 and NCT03350529.

**Supplementary Information:**

The online version contains supplementary material available at 10.1007/s13534-022-00250-y.

## Introduction

Many patients diagnosed with either localized prostate cancer (PCa) or benign prostatic hyperplasia (BPH) require treatment [1, 2]. MRI-guided transurethral ultrasound ablation (TULSA) is one emerging device that has been used to treat both diseases [3–6]. TULSA induces thermal coagulation through high-intensity ultrasound. Immediately after TULSA ablation, gadolinium-based contrast agents (GBCAs) are used to confirm the extent of ablation. The non-perfused volume (NPV) [7], which is calculated by measuring the absence of GBCA uptake in the prostate, is compared to the prescribed target volume. Substantial residual enhancing tissue inside the target volume is indicative of undertreatment.

While GBCAs are generally well-tolerated, they do have several downsides. First, they may accumulate in the brain and other body parts [8]. Second, patients with poor kidney function may also be ineligible [9]. Finally, during the high temperature exposures of thermal ultrasound, GBCAs may stay confined to the tissue-of-interest [10], potentially obscuring effects of subsequent ablations [7] and introducing local susceptibility artifacts [11]. If, during TULSA, undertreatment is determined based on post-treatment contrast-enhanced (CE)-imaging, follow-up treatment must be rescheduled several months later. This can lead to rising expenses and a negative impact on patient psychology [12]. It also necessitates the patient to undergo the entire treatment process once more, including renewed device instrumentation, meticulous bowel preparation, fasting and general anesthesia.

To avoid the use of GBCAs in the diagnostic setting, various researchers have used artificial intelligence (AI) to generate synthetic CE-images trained on contrast-free MRI sequences [13–16]. Most of these groups used the UNet architecture [17], a versatile model backbone used previously for both prostate segmentation [18–20] and prostate lesion identification [21], or a variant of Generative Adversarial Networks (GAN) [22]. Specific models included a basic 2D and 3D UNet, Fully Convolutional Network (FCN) and the Residual Vision Transformers (ResVit). Researchers were able to successfully generate accurate synthetic T1-weighted (T1w) CE-images from a variety of non-contrast MRI native T1w, T2-weighted (T2w), diffusion and susceptibility-weighted MRI sequences. Newer state-of-the-art models have also been used in medical image synthesis applications, largely consisting of GAN variants. Investigators have successfully synthesized various MRI contrast image types in the brain using the Collaborative GAN (CollaGAN) [23], ResVit [16], Adversarial Diffusion (Syndiff) [24] and Conditional GAN (cGAN) [25] models. Other successful applications with GAN-based techniques include synthesis of CT to MRI images in the male pelvis [16, 24], CT to PET images in the liver [26], and finally 3 T to 7 T MRI images in the brain [27].

In the context of TULSA therapy, an identical collection of both unenhanced and CE-MRI sequences is acquired during every treatment, a fact which can be leveraged for training a deep learning model to predict NPVs without contrast agents. First, a high-resolution T2w planning image is used to prescribe the treatment volume. Thereafter, real-time MRI thermometry is used to actively monitor the heating, both inside the prostate and around critical surrounding structures. Thermometry does have several limitations, such as sensitivity to air and patient motion [28], and in the case of TULSA, is a relative temperature technique, underscoring the need for post-treatment CE-imaging to assess the extent of coagulation. Accurate prediction of the final NPV during treatment could therefore allow immediate retreatment before it is too late and even obviate the need for GBCAs altogether.

The objective of the current study was to train a deep learning model using contrast-free, treatment-day MRI images acquired during TULSA therapy. The model outputted synthetic CE-images. The accuracy of these synthetic CE-images and corresponding NPV predictions was compared to ground truth to assess model performance.

## Materials and methods

### Source data

De-identified imaging data from a retrospective database of TULSA treatments was obtained from three separate clinical studies. All studies were conducted in accordance with the principles of the Declaration of Helsinki. Ethics approval was obtained for all studies and written informed consent was obtained. Ninety-five patients across four applicable patient groups were available:

(i) Whole-gland ablation for PCa (n = 64)
(ii) Partial ablation for PCa (n = 20)
(iii) Treat-and-resect after partial ablation for PCa (n = 5)
(iv) Partial ablation for BPH (n = 6)

Sixty-four (67%) patients received whole-gland prostate ablation, and thirty-one (33%) patients received partial ablation. No patient had missing imaging data. A flow participant diagram with inclusion and exclusion is demonstrated in Online Resource 1.

### Patient characteristics

Table 1 summarizes the patient baseline characteristics. All included patients were male and underwent TULSA as their first major prostate intervention. The majority of PCa patients had low- to intermediate-risk PCa, while BPH patients had moderate to severe symptom severity.

**Table 1:** Baseline patient characteristics

Median (IQR)				
Treatment intent	Age (years)	Prostate specific antigen (ng/ml)	Gleason score	IPSS
Prostate cancer (N = 89)	65 (58–69)	6.5 (5.0–9.1)	Gleason 6 (n = 26) Gleason 7 (n = 60) Gleason 8 (n = 3)	N.A
Benign prostatic hyperplasia (n = 6)	71 (65–72)	3.4 (2.7–3.7)	N.A	20 (16–27)

N.A Not applicable

### TULSA intervention

**Fig. 1:**
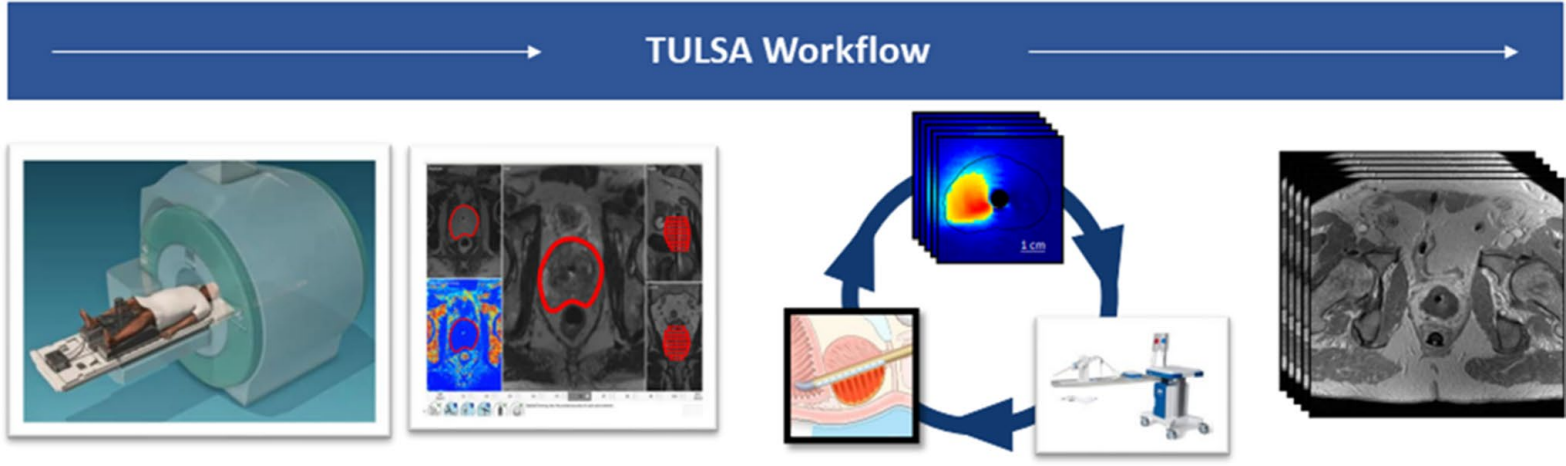
Description of MR-guided transurethral ultrasound ablation (TULSA) device. The transurethral ultrasound catheter ablates prostate tissue “inside out” to induce thermal coagulation. The therapy takes place fully inside the MRI suite, which enables accurate treatment planning and the use of MRI thermometry to monitor the heating. At the very end of treatment, MRI contrast agents are injected into the bloodstream, and the immediate non-perfused volume is compared to the prescribed treatment volume

TULSA (Profound Medical, Mississauga, Canada) is a class II medical device which is used to ablate prostate tissue. A detailed description of the TULSA intervention is described below (Fig. 1). The entire intervention took place in the MRI suite with the patient under general anesthesia, which enables physicians to accurately plan treatment volumes from high-resolution diagnostic MRI sequences. TULSA uses high intensity, spatially directed thermal ultrasound to coagulate prostate tissue. The therapeutic ultrasound catheter (22-French), which consists of a rigid brass rod with a plastic handle at the end, has ten individual ultrasound 4.5 × 5 mm elements located inside the rod with a small cut for the ultrasound to escape. Each ultrasound element can be controlled independently. The ultrasound catheter is fixated with an MRI-compatible robotic arm, which can perform both linear and rotational translation. The treatment is monitored using reference-based MRI thermometry, which allows physicians to monitor the heat deposition inside the prostate during the ablation, as well as around critical surrounding structures, such as the sphincter muscle, rectal wall, neurovascular bundles and bladder neck. Cooling water flowing through both the ultrasound catheter and a rectal device offer protection to the prostatic urethra and rectal wall.

Conformal ablation is achieved via a closed-loop control algorithm, which means ultrasound frequency and acoustic power, as well as rotation rate during the ablation, are fully automated. Dynamic, real-time thermometry images are acquired approximately every 5 s, with slices spanning the entire prostate, which provide a snapshot of the temperature distribution in and around the prostate. A typical treatment time is 40–60 min of ablation to treat the entire prostate gland. The treatment objective of the TULSA device is to achieve at least 240 cumulative equivalent minutes (CEM) at the prostate boundary, which is set by the manufacturer and cannot be adjusted. The accumulated thermal dose is significantly higher inside the prostate boundary however, since the ablation occurs inside-out. The control algorithm actively monitors the temperature distribution and will automatically adjust ultrasound outputs and rotation rate to achieve the most conformal ablation up to the treatment volume, but not exceeding it. If the operator wishes to achieve a higher thermal dose at the prostate boundary beyond 240 CEM, they can manually resweep the same target region multiple times. This approach was used several times by the TULSA operators to account for potentially varying thermal dose levels for different biological tissues.

### MR imaging protocol

All patients previously underwent the same TULSA treatment-day imaging protocol, which was conducted at 3 T on either Siemens (Prisma/Skryra, Erlangen, Germany) or Philips (Achieva/Ingenia, Best, Netherlands) scanners. Treatment planning was performed on a transverse T2w sequence. Ablation was monitored in real-time with a coaligned echo-planar imaging (EPI) sequence using both Magnitude (Mag) and Phase images. Phase images were then converted to temperature maps and thermal dose maps [29]. Two final temperature-based images were generated, representative of the whole treatment:

(i) A maximum temperature map (TMax), the maximum temperature achieved during treatment for each pixel
(ii) A thermal dose map (TDose), the cumulative thermal dose achieved during treatment for each pixel

Immediately after ablation, a native T1w scan without contrast was acquired followed by a T1w scan with contrast. A final subtraction image (Sub) was created by subtracting the native T1w image from the CE-T1w image. These Sub images from previously completed TULSA treatments are the ground truth images and represent the actual outcome from each treatment. Detailed sequence protocol information can be found in Online Resource 2.

### Data preparation

By design, all sequences (T2w, EPI and T1w) were already spatially coaligned, with a field-of-view of 256 × 256 mm, slice spacing of 5 mm, and 12 total slices. All images were then resampled to an in-plane resolution of 1 × 1 mm, and then center-cropped to 128 × 128 mm. T2w, T1w and Mag images were clipped to remove outliers, adjusted to have zero mean and variance of one, and then rescaled from 0 to 1. TMax images were clipped to a range of 35–85 °C, while TDose images were clipped to a range from 0 to 10,000 CEM, and then both were rescaled from 0 to 1. Only those slices where prostate was actively ablated were included. For each active slice, a corresponding physician-contoured prostate mask was generated from the prescribed ablation volume.

### Model description

**Fig. 2:**
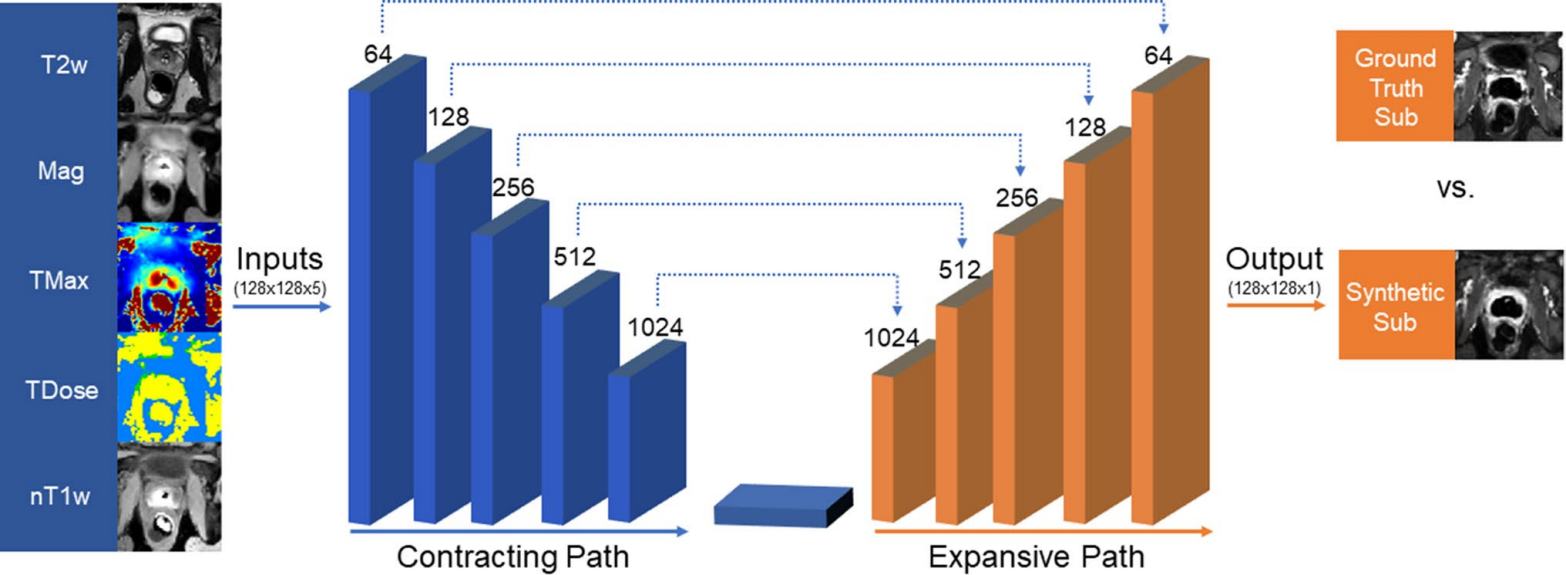
Modified 2D UNet convolutional neural network (CNN) architecture. Treatment-day, unenhanced MRI inputs included a T2-weighted (T2w) sequence, an echo-planar imaging-based thermometry scan used to monitor the ablation, and a native T1-weighted (T1w) acquired immediately after ablation. The thermometry scan was converted into three unique image types, including grayscale magnitude (Mag), the maximum temperature (TMax) and thermal dose (TDose). Ground truth subtraction images were used to train, validate, and test the model, which were calculated by performing a subtraction of the contrast-enhanced (CE)- and native-T1w images (nT1w). MRI inputs were then passed to a CNN, consisting of five down- and up-sampling blocks. For the contraction pathway, each block used three convolutional layers (kernel size of 3 × 3, stride of 2). After each convolutional layer, batch normalization was applied followed by a leaky rectified linear unit activation function. At the end of down-sampling block, max pooling with a size of 2 × 2 was used. At the bottom layer, three additional convolutional layers were built without max pooling. The convolution process was reversed until the original image input dimensions were obtained. Model output was a synthetic CE-enhanced subtraction image (Sub), which was compared to ground truth

The 2D deep learning UNet model was run on a Quadro P4000 NVIDIA GPU with CUDA Toolkit v11.2 and CuDNN SDK v8.1. The Tensorflow package was used for model training and testing. A description of the model architecture can be found in Fig. 2. Model inputs included five contrast-free, treatment-day MRI sequences including the T2w, TMax, TDose, Mag, and native T1w sequences. Model output was a synthetic Sub image, which was then compared to ground truth Sub image. Ground truth was the Sub image from the actual patient treatment that already occurred. The comparison of synthetic and ground truth Sub images, along with their corresponding NPV, indicates how strong the model prediction is. Quantitative analysis to assess image similarity is described later in more detail.

Allocation of train, validation and test data was approximately 80%/10%/10% and stratified to maintain a 2:1 proportion of whole-gland to partial ablation treatments. Train and validation datasets were used during model training, and the test dataset was used to assess model accuracy. The data was split as follows:

(i) Train: 75 patients with 2505 unique inputs and 501 unique outputs
(ii) Validation: 10 patients with 325 unique inputs and 65 unique outputs
(iii) Test: 10 patients with 360 unique inputs and 72 unique outputs

### Custom loss function

During model training, a custom loss function was created to minimize the difference between prediction and ground truth of Sub images. The custom loss function was a composite of three different functions including the mean absolute error (MAE), the structural similarity index (SSIM) and a prostate-weighted loss P1. MAE is the average absolute value pixel-by-pixel subtraction between ground truth and synthetic images. SSIM is a more complicated metric which takes into account perceived changes in image structural information, such as luminance and contrast. P1 loss was calculated in the same way as MAE, except pixels located outside the prostate mask were set to zero. During the initial testing phase, one of either the SSIM or MAE loss functions was used in isolation. While the initial results were promising, we attempted to further refine the accuracy of the model. This was achieved by adding the P1 loss and combining these three metrics into a composite loss function. Although the results of that ablation study are not reported here, the composite loss function produced the most accurate synthetic images compared to ground truth. The weighting scheme used by Chen et al. [15] was particularly effective, described in more detail below.

Each metric was weighted with individual coefficients ${\lambda }_{1}, {\lambda }_{2}$ and ${\lambda }_{3}$ and summed, described in Eq. (1).

1 $$custom\, loss = ({\lambda }_{1}*MAE)+{(\lambda }_{2}*(1-SSIM))+({\lambda }_{3}*P1)$$

For the first 40 epochs, ${\lambda }_{1}= {\lambda }_{2}={\lambda }_{3}=1$. For the next 40 epochs, the loss function was modified by setting ${\lambda }_{1}$ and ${\lambda }_{2}$ to 0.1 and ${\lambda }_{3}$ to 10, to force the model to focus on pixels inside the prostate. The epoch with the lowest recorded validation loss was taken as the final model. The Adam optimizer was used for all runs, with a learning rate of 1e−4 and a batch size of 12.

### Quantitative analysis

**Fig. 3:**
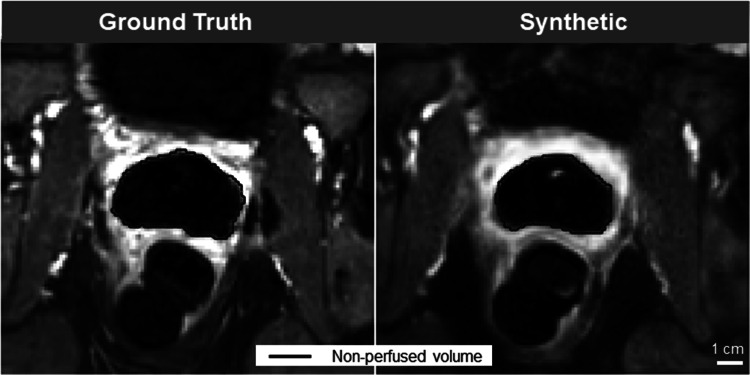
The non-perfused volume (NPV) is an important metric calculated at the end of a TULSA ablation, which gives the user immediate feedback on the ablation outcome. A hypointense void is surrounded by a bright rim of enhancement. A NPV that is considerably smaller than the target boundary is indicative of undertreatment. One metric used to evaluate model performance was the Dice-Similarity Coefficient (DSC). For all ground truth and synthetic images, the NPV was manually contoured, and the similarity calculated according to the DSC. A value of 100% represents a perfect score

Two types of quantitative analyses were executed when comparing synthetic to ground truth images:

(i) Synthetic image quality: Four quantitative metrics were used to evaluate image similarity including MAE, SSIM, as well as peak signal-to-noise ratio (PSNR) and the mean squared error (MSE). Quantitative comparison of ground truth vs. synthetic outputs of the CE-images was performed across both the entire image and a masked version of the same image. For the masked image, pixels inside the prostate kept their original value but outside were set to zero.
(ii) Accuracy of predicted NPV: NPV was manually segmented on both ground truth and synthetic images by author C.W. and verified by radiologist P.M. Segmentation was done blindly and randomly to avoid bias. Then, the Dice-Similarity Coefficient (DSC) was used to assess the accuracy of the NPV prediction. Example NPV segmentation is shown in Fig. 3.

Model accuracy as a function of prostate size, ablation type and slice location were also measured. The 95% confidence interval (CI) was calculated according to Conover [30]. All significant testing was performed using the non-parametric Wilcoxon rank-sum test.

### Sensitivity analysis

A sensitivity analysis was performed to determine which unenhanced MRI image type was the best NPV predictor. The model was thus retrained four times, excluding one or more inputs:

(i) No AXT1
(ii) No TMax
(iii) No TDose
(iv) Only T2w and Mag

### Qualitative assessment

A trained radiologist with over 5 years’ experience was asked for general feedback on the overall synthetic CE MRI image and NPV quality, which was compared directly to ground truth. Particular attention was given to the prostate, surrounding anatomy and the NPV. The radiologist was also asked to comment if the predicted NPV was indistinguishable from ground truth, and if the model tended to under- or overestimate the NPV. The ability of the model to predict any unintended heating outside the prostate was also assessed.

## Results

### Training performance

Training performance is summarized in Online Resource 3.

### Quantitative analysis

**Fig. 4:**
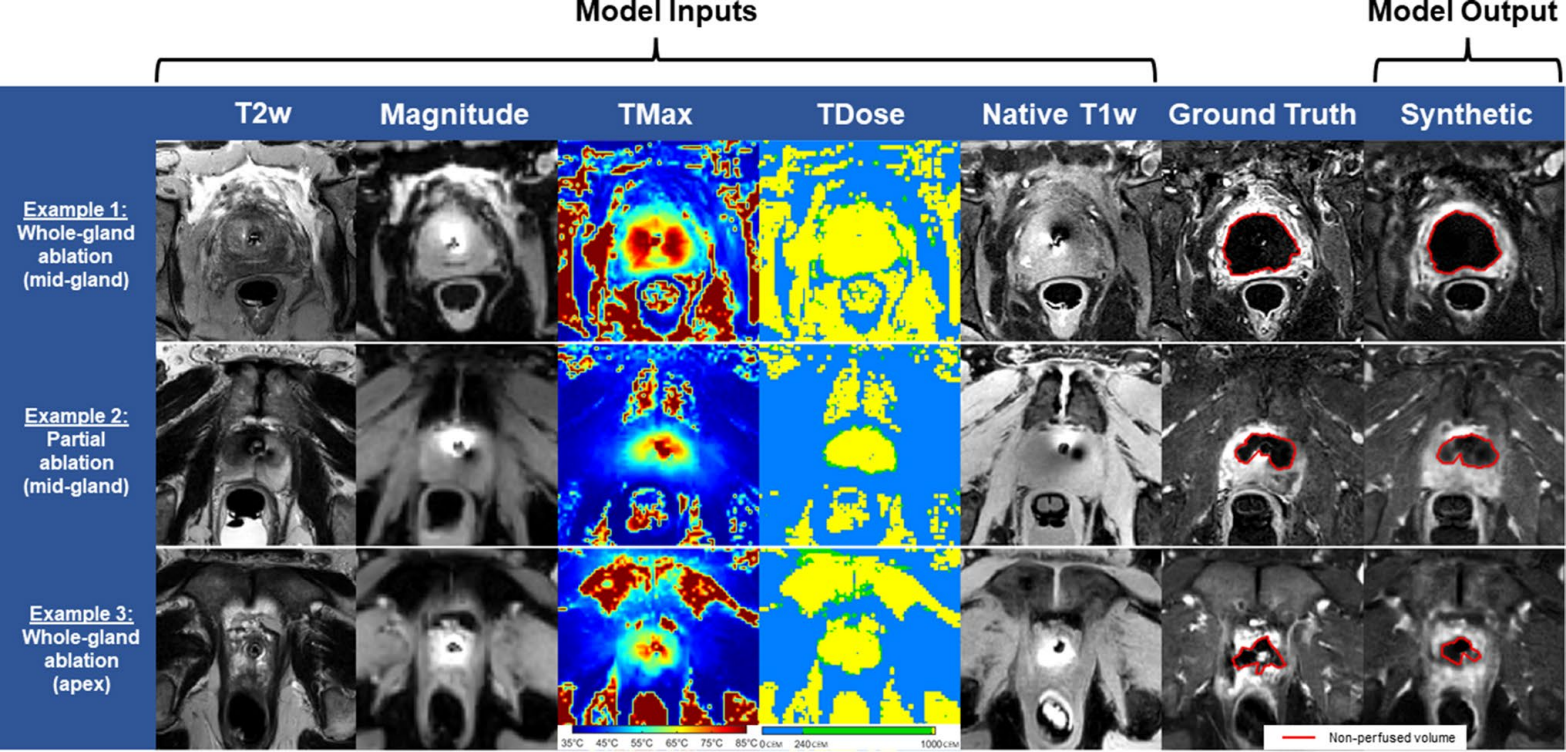
Model performance for different patient examples. For a mid-gland, whole-gland prostate cancer (PCa) ablation example (top row), the AI-predicted non-perfused volume (NPV) generated a score of 94%, measured according to the Dice-Similarity Coefficient (DSC) compared to ground truth. For a mid-gland, partial PCa ablation (middle row), the AI-predicted NPV scored 88% according to DSC. For the last whole-gland PCa ablation example (bottom row), in this case a slice located near the prostate apex where the prostate radius was smaller, a DSC score of 64% was reported

Figure 4 is a case example highlighting representative model inputs, outputs, and ground truths for three different test slices. In all cases, all five inputs were passed to the AI model. In the first whole-gland ablation example (top row), the AI-predicted NPV showed good agreement with ground truth for a mid-gland slice (DSC = 94%). For a partial ablation example (middle row), which was performed mid-gland, the AI-predicted NPV was correlated to ground truth (DSC = 88%). For the last whole-gland ablation example (bottom row), a slice located near the prostate apex, the AI model generated a DSC of 64%, indicative of weak similarity.

Table 2 summarizes the image similarity between ground truth and synthetic CE-images for all 72 slices in the test dataset. The performance across the whole image indicated a weak similarity, with a mean SSIM and MAE score of 0.34 and 0.14, respectively. Within the prostate, the mean SSIM and MAE was 0.93 and 0.02, respectively, indicating much closer agreement. The mean (std) DSC for the contoured NPV was 85% ± 8.1%, with 95% CI lower and upper bounds of 84% and 88%.

**Table 2:** Image similarity for test dataset, ground truth vs. synthetic

Mean (std.)				
	Structural similarity index (0 to 1)	Peak signal-to-noise ratio (dB)	Mean absolute error (0 to infinity)	Mean squared error (0 to infinity)
Whole image	0.34 ± 0.13	14.45 ± 2.18	0.14 ± 0.05	0.04 ± 0.02
Prostate only	0.93 ± 0.04	22.23 ± 2.51	0.02 ± 0.01	0.01 ± 0.004

The accuracy of the DSC was correlated to the size of the prostate radius, relative to the urethra center, performing significantly better (*p* < 0.001) when the maximum prostate radius was greater than 24 mm. Model performance was also significantly better on whole-gland compared to partial ablation slices (*p* < 0.001). Model performance approached significance (*p* = 0.051) for slices located mid-gland compared to the prostate apex and base.

### Sensitivity analysis

Model sensitivity to different training inputs and the resulting influence on predicted NPV is summarized in Table 3. The predicted NPV was closest to ground truth when trained with all five image inputs. Model performance was nearly unchanged when trained without the native T1w, TMax or TDose inputs, with a worst-case mean of 82.3%, with no significant differences detected amongst these four groups. Performance dropped significantly (*p* < 0.001) when the model was trained only with T2w and Mag inputs, with the mean (std) score dropping to 74.8% ± 13.6%.

**Table 3:** Dice-similarity coefficient of non-perfused volume—ground truth vs. synthetic images

	All inputs (%)	No native T1-weighted (%)	No maximum temperature (%)	No thermal dose (%)	Only T2-weighted and magnitude (%)
Mean	85.0	82.4	82.3	82.6	74.8
Std.	8.1	10.6	9.9	10.0	13.6
95% CI lower bound	84	82	82	82	71
95% CI higher bound	88	87	86	88	80

### Qualitative assessment

A qualitative assessment was performed by a trained radiologist for all test images. Overall, it was found that synthetic CE-images were blurrier than their ground truth counterparts. Synthetic CE-image quality was sub-optimal near the prostate apex and bladder neck.

Inside the prostate, the predicted NPV contour was more continuous, and smoother compared to ground truth. According to the radiologist, the predicted and ground truth NPV were indistinguishable for 22/72 (31%) of images, the majority located mid-gland, and deemed of sufficient quality that they could be confidently used to inform treatment decisions. The NPV was however under- and overestimated for 36/72 (50%) and 14/72 (19%) of images, respectively.

Outside the prostate, minor overshoot into the pelvic floor muscle occurred in 15 of 72 (20.0%) of ground truth test slices, which was correctly predicted 6 of 15 (40%) times by the AI model. While no patient had overshoot into the rectal wall on any ground truth test slices, the AI model incorrectly predicted overshoot on one single test slice, due to a rectal air bubble artifact that appeared on the TMax, TDose and native T1w images mid-treatment.

## Discussion

Using contrast-free MRI data from treatment-day TULSA treatments, realistic synthetic CE-images and accurate predictions of the NPV were generated by a deep learning model.

Across the entire image dataset, a mean SSIM and MAE of 0.34 and 0.14 was reported, respectively, which is less accurate than demonstrated in the brain [14–16]. During a TULSA procedure, the time between contrast-free imaging and GBCA administration is typically one hour to ninety minutes, which increases the risk of motion and misregistration. Moreover, the TULSA ablation causes acute coagulation necrosis in the prostate [31, 32] leading to complicated cellular, vascular, and inflammatory responses within and at the periphery of the thermal lesion [7], creating an NPV that is often disjointed and irregular in shape.

Despite these challenges, the deep learning model was able to accurately predict the NPV with a mean DSC score of 85%. For context, various researchers have examined the inter-operator variability of whole-gland prostate segmentation [20, 33] with reported DSC scores between 75 and 92%. Meanwhile, automatic prostate segmentation using modern deep learning algorithms have reported DSC scores of 87–92% [19, 20] compared to manual segmentation. Radiologist assessment indicated the deep learning model predicted an NPV that was indistinguishable from ground truth for roughly one third of slices, but also tended to underestimate the NPV. This is likely influenced by the custom loss function weighting, which warrants further investigation.

The deep learning model performed better at larger prostate radii (> 24 mm). This aligns with both manual and automatic segmentation techniques [19, 20, 33, 34], where higher variability was observed when contouring prostate zonal anatomy and slices near the prostate apex and base. This finding may partly be explained by the relationship between prostate size and the amount of thermal energy required to achieve coagulation [35]. To ablate further away, increased total thermal energy deposition is needed. Higher temperatures and thermal dose effectively increase the signal-to-noise ratio and increase the sharpness and size of the final NPV, simplifying the model’s task to detect correlations.

The sensitivity analysis offered additional insights. During TULSA ablation, clinical users rely on both the TMax and TDose to assess the coagulation extent in real-time. Discrepancies between TMax and TDose can occur, which may lead to user uncertainty. TDose is a composite of both temperature and time, while TMax is the highest recorded temperature. No statistical significance was observed when dropping TMax and TDose as inputs, leaving which image is a better NPV predictor unanswered. Furthermore, the native T1w did not have a significant impact on model performance. This suggests that real-time, predictive CE-image generation during thermometry monitoring is feasible, potentially allowing physicians to react even faster to any signs of undertreatment.

The radiologist noted that synthetic images tended to be blurrier, which has also been reported elsewhere [14, 15]. It was noted that the predicted NPV was generally smoother and anatomical features near the bladder neck and prostate apex were more difficult to resolve than mid-gland, consistent with the quantitative analysis. Several included patients were not catheterized or had inefficient suprapubic catheter drainage. Dynamic bladder filling likely lead to discrepancies between the planning, thermometry and final CE-images, particularly near the bladder neck. Outside the prostate, our model correctly predicted NPV extending into the pelvic floor 40% of the time. Unintended heating outside the prostate can impact the TULSA safety profile, and if deemed clinically important, the model could be optimized for both the prostate and pelvic floor muscles, through small adjustments to the custom loss function.

Since the current work was geared towards establishing feasibility, we opted for the well-established and straightforward 2D UNet model backbone. Yet it was commonly observed that within the same patient, certain cross-sectional slices had worse quantitative performance than others. 3D CNNs can outperform 2D CNNs [15, 36] which could rectify this problem. On the other hand, 3D CNN’s have only marginal performance benefits and introduce increased model complexity, longer training times and large memory requirements. Traditional CNN models such as UNet may also suffer from undesirable loss of detailed structure such as irregular anatomy [16, 25]. Repeating the present work with state-of-the-art GAN-based variants, such as the CollaGAN, ResVit, SynDiff or cGAN, which have been shown to better capture higher spatial resolution information due to the use of adversarial loss [25], could greatly improve accuracy of the post-TULSA NPV predictions. This is particularly relevant when one considers that the post-TULSA NPV is often irregular or discontinuous.

There are several limitations in this study. The need for large amounts of clinical data is essential for deep learning, and we included less than one hundred patients, which was all that was available. Many disqualifications occurred due to patient motion and unintended peristaltic motion causing gas bubbles in the rectum, despite the routine use of general anesthesia, meticulous bowel preparation, fasting and anti-peristaltic medication during therapy. Patient motion caused challenging registration, which was not addressed. Gas bubbles, on the other hand, caused large “blooming” thermometry artifacts, which often extended into the prostate [37], limiting its usefulness as a prediction sequence. Additionally, only one radiologist performed a qualitative assessment of synthetic image quality, meaning intra-observer variability could not evaluated. This omission was rationalized due to the fact this investigation was geared towards establishing feasibility. While data augmentation was not found to increase model performance during initial feasibility testing, it may play a role in recovering “lost” datasets through creation of simulated artifacts that the model could potentially account for. Another limitation is that the prostate mask was manually contoured, which was necessary to train and then later evaluate the deep learning model. Manual contouring is however labor-intensive and inefficient. Not only would automatic prostate segmentation simplify the training and evaluation of the deep learning model, it could also help streamline the overall treatment-day TULSA workflow.

To summarize, we have demonstrated that by using treatment-day, contrast-free MRI sequences from TULSA treatment including T2w, EPI and native T1w sequences, one can generate synthetic CE-images with predicted NPVs close to ground truth. Refinement of this technique can be achieved with a larger training set. If developed further, it is hoped that this technique will give treating physicians an opportunity to optimize treatment outcomes within a single treatment session, and potentially obviate the need for GBCAs altogether.

## Supplementary Information

Below is the link to the electronic supplementary material.

Supplementary file1 Flow participant diagram. Ninety-five patients in total were included, who were treated for a range of diseases including prostate cancer (PCa) and benign prostatic hyperplasia (BPH). (TIF 82 KB)

Supplementary file2 MRI sequence information for both model input and ground truth, including weighted (T2w), T1-weighted (T1w) and Echo Planar Imaging (EPI) sequences. The EPI sequence formed the basis of the MRI thermometry sequence, which was converted to a magnitude image, a maximum temperature map and a thermal dose map. (TIF 96 KB)

Supplementary file3 Training performance for all five model inputs (T2w, Mag, TMax, TDose, native T1w). Model training lasted approximately 43 minutes on a Quadro P4000 NVIDIA GPU, needing 61 epochs in total. After the second 40-epoch run with the modified custom loss function (λ_1_= λ_2_=0.1, λ_3_=10), train/validation/test loss were recorded for the best model run. Results are summarized in the table below. (TIF 53 KB)
