# Supplementary figures and images for: Deep learning prediction of non-perfused volume without contrast agents during prostate ablation therapy
Source: Biomed Eng Lett. 2022 Nov 8;13(1):31–40. doi: 10.1007/s13534-022-00250-y (PMC9873841; doi:10.1007/s13534-022-00250-y)

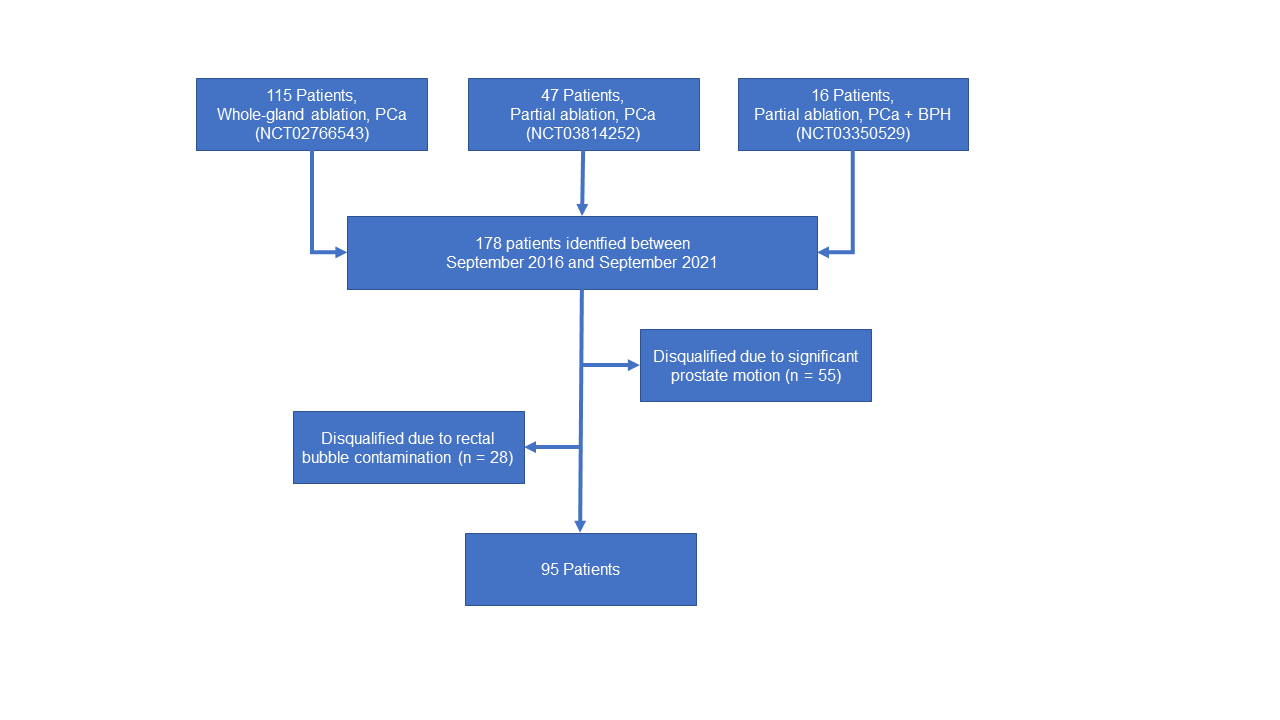

Supplement: Supplementary file 1 — Supplementary file1 Flow participant diagram. Ninety-five patients in total were included, who were treated for a range of diseases including prostate cancer (PCa) and benign prostatic hyperplasia (BPH). (TIF 82 KB) [file 13534_2022_250_MOESM1_ESM.tif]

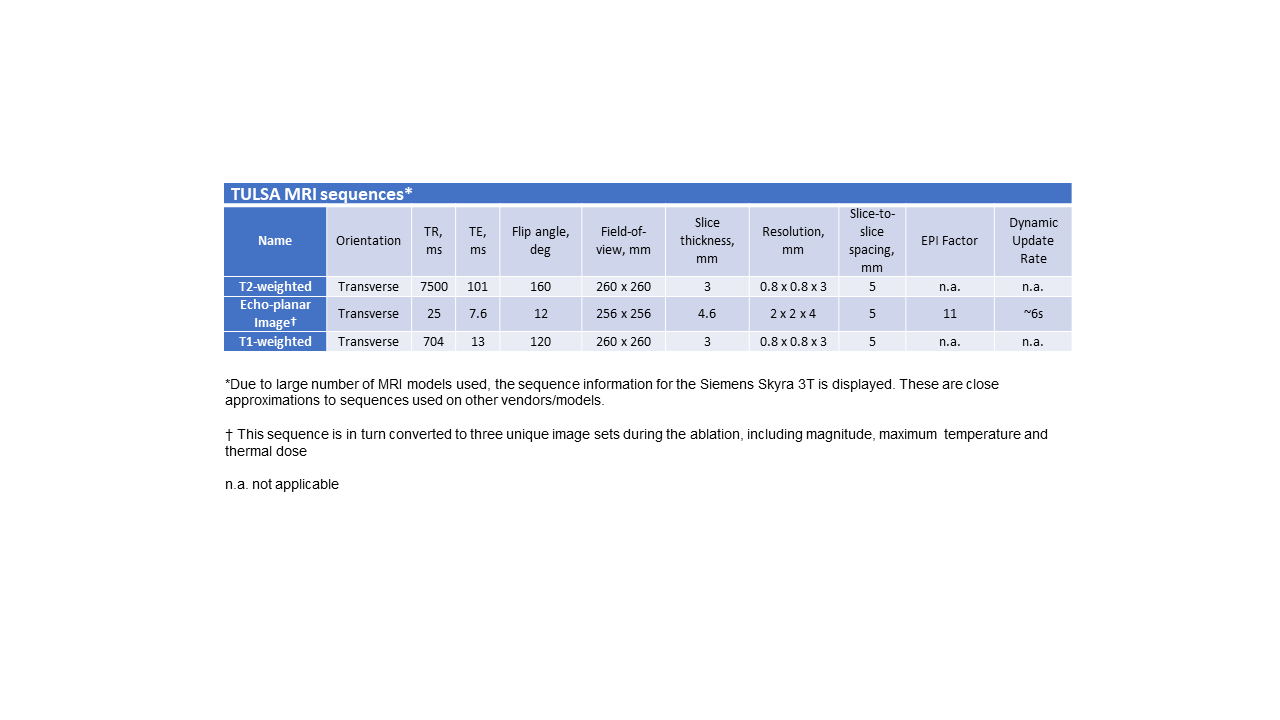

Supplement: Supplementary file 2 — Supplementary file2 MRI sequence information for both model input and ground truth, including weighted (T2w), T1-weighted (T1w) and Echo Planar Imaging (EPI) sequences. The EPI sequence formed the basis of the MRI thermometry sequence, which was converted to a magnitude image, a maximum temperature map and a thermal dose map. (TIF 96 KB) [file 13534_2022_250_MOESM2_ESM.tif]

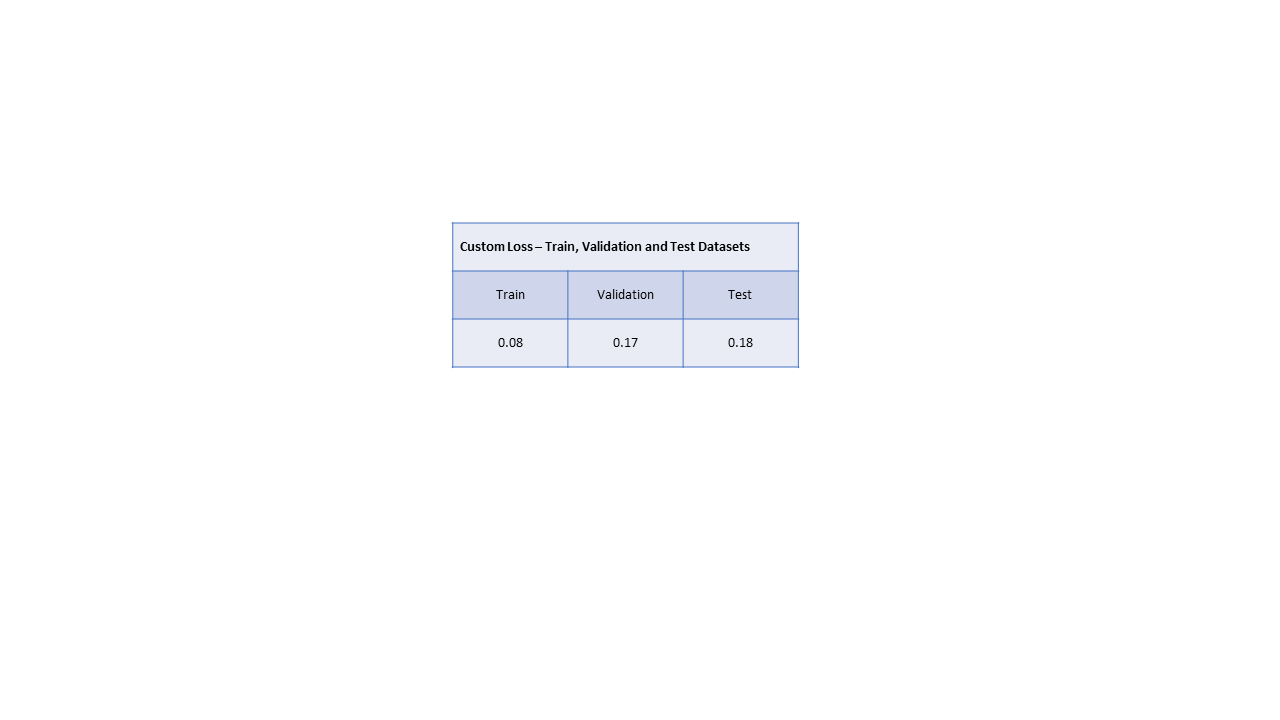

Supplement: Supplementary file 3 — Supplementary file3 Training performance for all five model inputs (T2w, Mag, TMax, TDose, native T1w). Model training lasted approximately 43 minutes on a Quadro P4000 NVIDIA GPU, needing 61 epochs in total. After the second 40-epoch run with the modified custom loss function (λ1= λ2=0.1, λ3=10), train/validation/test loss were recorded for the best model run. Results are summarized in the table below. (TIF 53 KB) [file 13534_2022_250_MOESM3_ESM.tif]
